# Nicaragua’s human rights crisis requires international response

**DOI:** 10.1371/journal.pntd.0007119

**Published:** 2019-03-21

**Authors:** Jorge A. Huete-Pérez

**Affiliations:** University of Central America, UCA, Managua, Nicaragua; Saudi Ministry of Health, SAUDI ARABIA

An eight-year-old child died last August in Nicaragua from dengue fever, one of several neglected tropical diseases (NTDs) plaguing this Central American nation, despite persistent national and international efforts to eradicate the disease. What makes this child’s death even more painful is that it was probably avoidable had there been better medical attention available. Since April 19 of this year, a crisis of ungovernability exploded, seemingly overnight. Unprecedented acts of government violence and repression have led to a widespread human rights crisis with disastrous repercussions for the society, particularly in the economic, educational, and health sectors. Close to 300 doctors and medical professionals have been arbitrarily fired by Daniel Ortega’s government in reprisal for speaking out against government oppression, refusing to attend government mandated marches, and, most importantly, for treating those wounded by government official and extra-official forces, including paid gangs and paramilitaries. Some of those doctors were specialists in areas such as NTDs.

Years of abuse by an increasingly oppressive regime have gradually crushed all democratic institutions and provided the kindling for what has become a nationwide inferno, sparked by the government’s violent response to peaceful student protests against social security reforms. For the past eight months, government repression has been responsible for illegal detentions; extrajudicial executions; disappearances; cruel and degrading treatment of detainees including torture; censorship of the press; and threats, physical attacks, and imprisonment of medical professionals. According to multiple Nicaraguan and international human rights organizations (including Inter American Commission on Human Rights [IACHR], Office of the United Nations High Commissioner for Human Rights, Amnesty International, Human Rights Watch, International Federation of Human Rights, and others), the Nicaraguan government, police, and paramilitary forces are responsible for the violence. The current death toll ranges between 300 and 500 with thousands wounded; there are close to 300 political prisoners and an uncertain number of citizens who have disappeared [1].

The Nicaraguan government’s response to peaceful civilian protests violates multilateral obligations under international law, including the American Convention on Human Rights and the International Covenant on Civil and Political Rights, guaranteeing right to life, liberty, freedom of speech, and free assembly.

**Fig 1:**
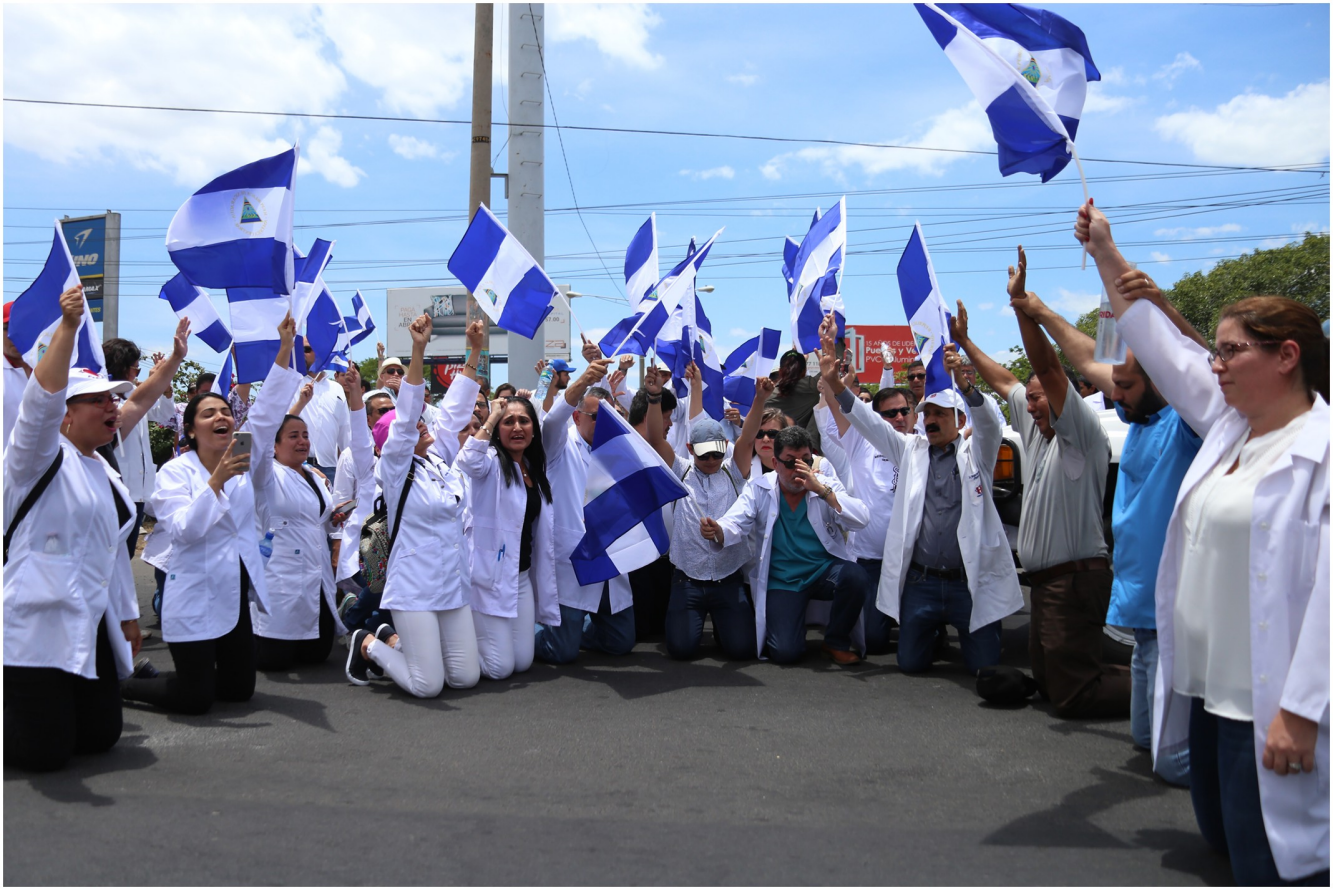
In the context of social protest in Nicaragua, hundreds of academics and healthcare professionals have protested in the streets against dismissals, persecution, and criminalization of medical personnel. Picture by Orlando Valenzuela, El Nuevo Diario (Permission granted).

It is well documented by the Nicaraguan Medical Association (ANM) and others that more than 300 medical professionals have been threatened, fired, and jailed for ignoring Nicaraguan government orders to refuse treatment of those wounded in protests (Fig 1). National and international human rights organizations have denounced this denial of healthcare and have expressed outrage over government interference in medical personnel adhering to their Hippocratic Oaths. In June, the World Medical Association condemned the collapse of Nicaragua’s healthcare system, even prior to the mass firings, death, and imprisonment of medical personnel.

Most police and paramilitary attacks have taken place on or around university campuses and roadblocks set up by opposition activists throughout the country. The roadblocks were removed by the government by force in mid-July, resulting in a heavy death toll. Public university personnel who have spoken out against such attacks have been fired, yet the Nicaraguan Constitution guarantees autonomy for institutions of higher education and the inviolability and immunity of university campuses as fundamental principles of the national system of higher education.

The erosion of safety and security within Nicaraguan universities and medical centers has had a disastrous impact on scientific research, education, and medical services with far-reaching consequences for Nicaraguan society.

Although the early incidents of violent government repression have ceased, the government is now directing more surreptitious attacks on medical personnel, students, academicians, and intellectuals, using a hastily drafted “antiterrorism” law to eliminate and punish all forms of dissent.

Protests against state brutality and repression are now carried out by all sectors of society, most notably university students; mothers of those imprisoned, murdered or disappeared; and medical personal fired from state health institutions. The government is responding with house-to-house raids and search and seizures of outspoken opposition members at work, at marches, on public streets, and on public transportation. Universities, including the National Autonomous University of Nicaragua—Leon, Medical School, which was closed for five months, were pressured by the government to reopen. Most international collaborative research projects, however, have either been cancelled or postponed. Students are leaving the country in droves. Four medical doctors and four medical students have now been shot and killed by government forces, one of whom was a young Brazilian completing her residency. Another medical student, a Belgian national, is currently being held in the infamous El Chipote jail awaiting trial on charges of “terrorism.”

The arbitrary firing of specialized doctors from public institutions, some of whom are the only medical personnel in Nicaragua trained in NTDs, is already having a disastrous impact on the nation’s healthcare system, particularly in Leon where more than 80 of the 300 doctors who have been fired thus far were located. They include specialists trained internationally at prestigious universities in the United States and Europe—such as University of North Carolina, Vanderbilt University, University of Texas, University of Zaragoza, and Karolinska Institute—at much cost to the nation and the host institutions. The firings have led to the cancelation of several research and exchange collaborations on infectious disease with US universities, such as University of Virginia and University of North Carolina. Nicaragua is especially vulnerable to NTDs, with intestinal helminth infections and vector-borne NTDs such as Chagas disease, leishmaniasis, vivax malaria, and dengue being the leading NTD concerns [2].

NTDs have a greater impact on populations already made vulnerable by malnutrition and limited access to quality health services. Nicaragua has the ignominious distinction of being the second poorest nation in the Americas, as well as having the second highest level of undernourishment. According to the study "Food security and nutrition in the world" by the Food and Agriculture Organization of the United Nations (FAO) and other agencies, Bolivia has the highest percentage of citizens suffering from food insecurity, with 19.8% of its population undernourished, followed by Nicaragua at 16.2% [3]. By further reducing the number of specialized health providers in clinics and teaching hospitals for an already compromised population, the government is opening the door to more frequent and profound outbreaks of NTDs in this nation of over 6 million people.

International donors of collaborative research and medical cooperation are freezing or withdrawing funding. Medical doctors and academicians are becoming increasingly outspoken, however, the most vocal being the presidents of the University of Central America (UCA), the American University (UAM), and the Nicaraguan Medical Association (AMN). The two former have received death threats. Speaking out in support of students and academic freedom, the Academy of Sciences of Nicaragua has issued many declarations expressing deep concern, rejecting “all violations of the rights of freedom of expression and freedom of assembly.” The Academy’s president has also received death threats and has gone into exile.

The deepening humanitarian crisis, economic uncertainty generated by the predicted collapse of the economy (the Nicaraguan Central Bank has stopped publishing its daily financial reports), and intensified perfidious violence have jettisoned cross-border migration to levels seen only during the Contra War of the 1980s and have prompted the exodus of more than 23,000 citizens to Costa Rica alone, drawing neighboring countries into the conflict. The likely collapse of the economy, should the situation not improve within a few months, has prompted the government to cut key areas of the national budget, including health, education, and the environment.

As the conflict reaches its fifth month, we beseech the international medical community to join the International Human Rights Network of Academies and Scholarly Societies in petitioning the Nicaraguan government “to put an end to the violence, arbitrary detention, threats and harassment used to inhibit individuals from expressing their views on social issues” [4]. It is paramount that medical professionals, scientists, human rights advocates, and the global academic fora in general share knowledge, voice concerns, and demand an independent, transparent investigation into human rights abuses in Nicaragua. Vulnerable Nicaraguan doctors, intellectuals, and universities need institutions abroad to provide outspoken global solidarity to prevent further violence and death. International organizations lending their support and expertise in preventative diplomacy are critical to finding a peaceful and democratic resolution to this explosive situation. During the postconflict era, their support will be needed to rebuild democratic institutions and restore the rule of law. Nicaragua will also need support from the global medical community to repair the damage done to the Nicaraguan health sector’s infrastructure, with additional training programs to replace specialists in NTDs who were forced to flee government repression and imprisonment.

We are asking the global community to end tolerance for continued bloodshed in Nicaragua. Given the propensity for violent conflict in Nicaragua and the region, the failure of the attempted national dialogues and the iron-fisted control of all state institutions by President Ortega, the international community is vital to preventing Nicaragua from plunging into another civil war.
